# High Meat Intake and Ferritin Levels in Relation to Cardiovascular Risk Among Individuals with Diabetes in Mongolia

**DOI:** 10.3390/nu16234245

**Published:** 2024-12-09

**Authors:** Narkhajid Galsanjigmed, Munkhuchral Nordog, Altaisaikhan Khasag, Odgerel Tsogbadrakh, Oyuntugs Byambasukh, Otgonbat Altangerel

**Affiliations:** 1 Department of Endocrinology, SOM, Mongolian National University of Medical Sciences, Ulaanbaatar 14210, Mongolia; narkhajid@mnums.edu.mn (N.G.); altaisaikhan@mnums.edu.mn (A.K.); 2 Department of Hematology, SOM, Mongolian National University of Medical Sciences, Ulaanbaatar 14210, Mongolia; munkhuchral@mnums.edu.mn (M.N.); odgerel.ts@mnums.edu.mn (O.T.)

**Keywords:** meat intake, ferritin, cardiovascular risk, diabetes, Mongolia

## Abstract

Background/Objectives: Mongolian diets are characterized by high meat consumption, which may contribute to dietary iron intake and influence ferritin levels and cardiovascular risk. Elevated ferritin levels have been associated with inflammation and cardiovascular disease (CVD) risk in various populations; however, the specific effects of high meat intake and ferritin levels on CVD risk in Mongolian individuals with diabetes remain unclear. This study aimed to assess the relationship between meat intake, ferritin levels, and cardiovascular risk markers in a diabetic Mongolian population. Methods: A cross-sectional study was conducted involving 171 Mongolian adults with diabetes. Meat intake was assessed using 24 h dietary recall interviews, and participants were categorized into tertiles of low, medium, and high intake. Blood samples were collected to measure ferritin, lipid profiles, and other CVD markers. The Framingham Risk Score was calculated for each participant. Results: Participants in the highest tertile of meat intake exhibited significantly elevated ferritin levels compared to those in the lower tertiles (275.6 ng/mL vs. 119.6 ng/mL, *p* = 0.001). Elevated ferritin levels were observed in 40% of participants and were associated with higher LDL cholesterol (3.75 vs. 3.22 mmol/L, *p* = 0.002), total cholesterol (5.63 vs. 5.2 mmol/L, *p* = 0.012), and Framingham Risk Scores (13.97 vs. 11.4, *p* = 0.0001). However, ferritin levels showed no significant association with other cardiovascular or inflammatory markers, including BMI, HbA1c, CRP, and IL-6 (*p* > 0.05). Mediation analysis revealed that ferritin partially mediated the relationship between meat intake and cardiovascular risk (beta coefficient = 0.539, *p* = 0.001), though the indirect effect was not statistically significant. Interaction analysis indicated no significant effect modification of meat intake and ferritin levels on cardiovascular risk (*p* = 0.844). Conclusions: In this diabetic Mongolian population, high meat intake was associated with elevated ferritin levels, which may have reflected dietary iron intake rather than systemic inflammation or increased CVD risk.

## 1. Introduction

Mongolian diets are heavily influenced by the country’s harsh climate and nomadic lifestyle, leading to a high reliance on red meat, especially from cattle, sheep, and goats [1,2]. This high meat intake provides essential nutrients such as protein, fat, and iron, which are necessary for survival in cold climates but may also affect health markers in unique ways [3]. Elevated meat consumption has been associated with various health risks, including increased risk of cardiovascular disease (CVD), diabetes, and certain cancers [4,5]. Among the specific markers linked to high red meat intake, ferritin—a protein that stores iron—is often elevated due to increased iron from dietary sources, particularly meat [6,7].

Ferritin is not only an indicator of iron storage but also a biomarker of systemic inflammation and oxidative stress, especially in individuals with metabolic disorders such as diabetes [8,9]. Elevated ferritin levels are frequently associated with metabolic syndrome, insulin resistance, and dyslipidemia, which contribute to CVD risk [10,11,12]. However, these relationships may differ in populations with distinct dietary patterns, where high iron intake from meat could elevate ferritin without necessarily increasing inflammation or CVD risk. Studies in Inuit populations, who also consume high amounts of animal-based foods, suggest that unique metabolic adaptations might mitigate the negative effects of high-fat, high-protein diets on cardiovascular health [13,14]. Given these adaptations, it is possible that Mongolians exhibit metabolic adjustments that influence how ferritin, lipids, and other CVD markers respond to high meat intake.

This study aimed to investigate the association between meat intake, ferritin levels, and cardiovascular risk markers in a diabetic Mongolian population. We hypothesized that while high meat intake would correlate with elevated ferritin levels, ferritin may not strongly predict CVD risk markers in this unique population, highlighting the potential need for culturally adapted health guidelines.

## 2. Materials and Methods

### 2.1. Study Design and Population

This cross-sectional study included 171 diabetic Mongolian adults. Inclusion criteria required participants to have a confirmed diagnosis of type 2 diabetes, be aged 30–75 years, and provide informed consent. Exclusion criteria included recent changes in dietary habits, use of iron supplements, renal or hepatic failure, cancer, and excessive alcohol consumption, as these factors could influence ferritin levels [15].

This study was conducted in compliance with the principles of the Helsinki Declaration and approved by the Medical Ethics Committee of the Mongolian National University of Medical Sciences University (approval code: 22/Z-08, 23 September 2022).

### 2.2. Dietary Assessment: 24 h Dietary Recall

Meat intake was assessed using a 24 h dietary recall method, in which trained nutritionists conducted structured interviews with each participant. Participants recalled all foods and beverages consumed in the previous 24 h, detailing portion sizes, preparation methods, and accompaniments [16]. The 24 h dietary recall method was used as it provides a reliable and practical method for estimating short-term dietary intake in large-scale studies. Participants were guided through their recall using standardized portion size assessment tools to ensure accuracy.

Each participant’s average daily meat intake was calculated in grams using a national food composition database. Participants were then categorized into tertiles based on meat intake: low (bottom one-third), middle (middle one-third), and high (top one-third). This classification enabled us to examine differences in health markers across varying levels of meat consumption. Interviewer training, multiple recall days, and data cleaning procedures were implemented to ensure data accuracy. Outliers and discrepancies were flagged and verified with participants when possible. These steps helped standardize dietary intake data and minimize reporting bias.

### 2.3. Ferritin and Health Marker Assessment

Blood samples were collected from participants following an overnight fast. Serum ferritin was measured using enzyme-linked immunosorbent assay (ELISA) methods. Ferritin levels were categorized as “normal” or “elevated” according to clinical cutoffs. Additional health markers, including lipid profiles (total cholesterol, HDL, LDL, triglycerides), HbA1c, and inflammatory markers (CRP and IL-6), were measured. Ferritin and inflammatory markers (CRP, IL-6) were measured using ELISA kits (Ferritin ELISA, DRG International, Springfield, NJ, USA; CRP and IL-6 ELISA, Thermo Fisher Scientific, Waltham, MA, USA) and analyzed using a SpectraMax iD3 plate reader (Molecular Devices, San Jose, CA, USA). Clinical cutoffs for ferritin were set at ≥150 ng/mL for women and ≥300 ng/mL for men. In addition to lipid and inflammatory markers, soluble transferrin receptor (sTFR), reticulocyte hemoglobin equivalent (RET-He), and homocysteine levels were analyzed. sTFR and RET-He were measured using enzyme-linked immunosorbent assay (ELISA) kits (manufacturer: XYZ Diagnostics, Lakeway, TX, USA) and analyzed with a SpectraMax iD3 plate reader (Molecular Devices, San Jose, CA, USA). Homocysteine was quantified using high-performance liquid chromatography (HPLC) with fluorescence detection. Assays were conducted following the manufacturer’s protocols to ensure accuracy. Furthermore, blood pressure was measured using an automated sphygmomanometer and BMI was calculated from height and weight measurements.

The Framingham Risk Score (FRS) was calculated for each participant as an estimate of their 10-year cardiovascular risk. This validated tool incorporates age, gender, cholesterol levels, blood pressure, smoking status, and diabetes status [17].

### 2.4. Statistical Analysis

The characteristics of the study population were expressed as means with standard deviations (SDs) for continuous variables and as percentages for categorical variables, stratified by meat intake and ferritin categories. Differences in health markers across meat intake tertiles and ferritin categories were assessed using one-way ANOVA or chi-square tests. Pearson correlation coefficients examined associations between continuous variables such as meat intake and ferritin levels.

Mediation analysis was conducted to explore whether ferritin levels mediated the relationship between meat intake and CVD risk, represented by the FRS. The mediation pathway included the following: (1) Path A tested the association between meat intake and ferritin levels; (2) Path B tested the association between ferritin levels and FRS. The significance of the indirect effect was evaluated using regression analyses.

Interaction analysis was performed to evaluate whether the relationship between meat intake and FRS differed by ferritin levels. An interaction term (“meat_intake * ferritin”) was created and included in the logistic regression models. All models were adjusted for age, sex, BMI, and inflammatory markers (CRP, IL-6).

Statistical analyses were performed using IBM SPSS V.28.0 and graphical representations were generated using GraphPad Prism 9.0. A *p*-value < 0.05 was considered statistically significant for all analyses.

## 3. Results

The mean age of the participants was 56.7 years, with no significant difference across the tertiles of meat intake (*p* = 0.24). The gender distribution was relatively balanced across the groups, with around 36.8% of the participants being male. No significant differences were observed between the groups regarding gender distribution (*p* = 0.984). A majority of participants (60.3%) had an education level below a bachelor’s degree, with no statistically significant differences across meat intake categories (*p* = 0.245). The majority of participants were married or cohabiting (76.6%), and, although there was a trend toward higher marriage or cohabitation rates in the middle meat intake group (83.1%), this difference was not statistically significant (*p* = 0.218). Smoking and alcohol consumption did not significantly differ across the meat intake groups (*p* = 0.814 and *p* = 0.114, respectively). However, alcohol consumption showed a trend toward being higher in the highest meat intake group (41.2%) compared to the lowest (26.1%) (*p* = 0.05 for T2 vs. T3). Participants were asked whether they adhered to a diet and physical activity recommended for diabetes management. Those who answered “no” (56.0% and 35.3%) indicated that they were not following dietary and physical activity recommendations despite their condition. The mean duration of diabetes was 9.63 years, with no significant differences observed between the tertiles of meat intake (*p* = 0.317, Table 1).

As shown in Table 2, mean BMI and blood pressure values (systolic and diastolic) were also similar across the groups, with no significant variations. Meat intake was significantly associated with ferritin levels, with the highest meat intake group showing the highest mean ferritin levels (275.6 ng/mL vs. 119.6 ng/mL in the lowest intake group, *p* = 0.001). Additionally, meat intake was associated with higher LDL cholesterol levels, although this difference was not statistically significant. No differences were observed in other biomarkers, such as HbA1c, total cholesterol, HDL cholesterol, and triglycerides, across the groups. Inflammatory markers, including IL-6 and CRP, showed no significant differences across meat intake categories. Similarly, no significant differences were observed in the Total Framingham Risk Score between the tertiles of meat intake, suggesting that higher meat intake might not be associated with increased cardiovascular risk in this population.

**Figure 1:**
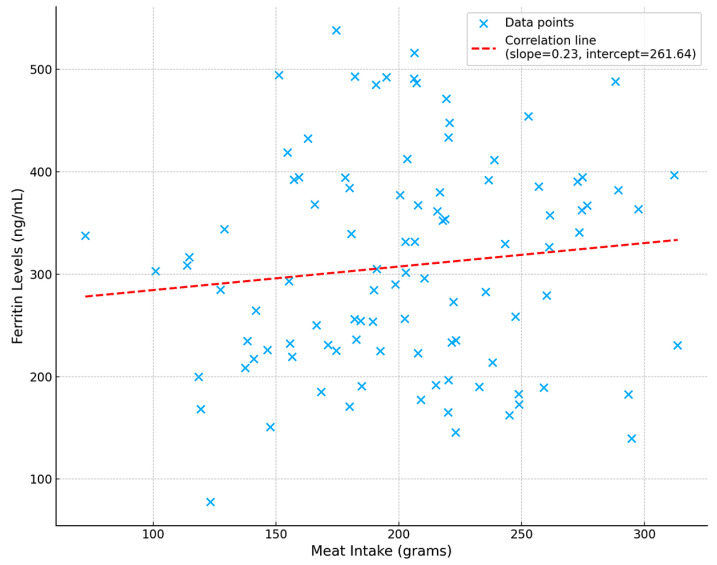
Relationship between meat intake and ferritin levels.

Participants in the highest meat intake tertile exhibited the highest average ferritin levels. Table 2 displays ferritin levels across the tertiles of meat intake and Figure 1 provides a scatterplot of meat intake versus ferritin levels, showing a gradual increase in ferritin as meat intake increased. There was a positive correlation between meat intake and ferritin levels (r = 0.25, *p* = 0.041), with mean ferritin levels increasing across the tertiles of meat intake.

Among the participants, 60% had normal ferritin levels, while 40% exhibited elevated levels. Elevated ferritin was more common among females compared to males (*p* = 0.0001), as shown in Table 3. Other demographic and lifestyle factors, including age, education level, smoking status, alcohol use, and physical activity, showed no significant differences between the normal and elevated ferritin groups.

Elevated ferritin was also associated with higher LDL cholesterol (3.75 vs. 3.22 mmol/L, *p* = 0.002) and total cholesterol (5.63 vs. 5.2 mmol/L, *p* = 0.012), as presented in Table 2. Additionally, participants with elevated ferritin had higher Framingham Risk Scores (13.97 vs. 11.4, *p* = 0.0001). However, no significant differences were observed for other cardiovascular risk markers and inflammatory markers.

Mediation Analysis: Meat intake was significantly associated with ferritin levels (beta coefficient = 0.539, *p* = 0.001), and ferritin levels were significantly associated with FRS (OR = 2.279, *p* = 0.041). However, the indirect effect of meat intake on FRS through ferritin was not significant, suggesting partial mediation.

**Figure 2:**
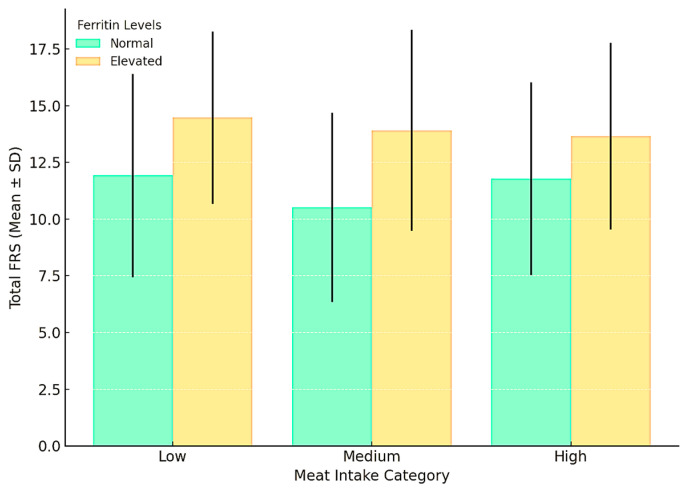
Interaction between meat intake and ferritin levels on CVD risk, represented by the FRS. Bars represent mean ± SD. Elevated ferritin levels were associated with higher FRS scores across all meat intake categories. However, no significant interaction effect was observed.

Interaction Analysis: The interaction term between meat intake and ferritin levels was not statistically significant (*p* = 0.844), indicating no significant effect modification. As shown in Figure 2, FRS scores were consistently higher in the elevated ferritin group across all meat intake categories. However, no significant interaction was detected between ferritin levels and meat intake (Figure 2).

## 4. Discussion

This study provides novel insights into the relationship between high meat intake, ferritin levels, and cardiovascular risk in a diabetic Mongolian population. First, we observed a significant positive association between meat intake and ferritin levels, consistent with known mechanisms of dietary iron absorption. This finding suggests that high meat consumption in Mongolian diets contributes to elevated ferritin levels, which may reflect dietary iron intake rather than systemic inflammation. Second, elevated ferritin levels were independently associated with higher LDL cholesterol and Framingham Risk Scores (FRS), supporting its potential role as a biomarker of lipid metabolism and cardiovascular health. However, no significant associations were found between ferritin levels and traditional inflammatory markers such as CRP and IL-6, indicating that ferritin’s role in cardiovascular risk may operate through non-inflammatory pathways. Finally, mediation analysis revealed that while ferritin partially mediated the relationship between meat intake and FRS, the indirect effect was not statistically significant. This suggests that additional mechanisms, such as oxidative stress or metabolic dysregulation, may also play a role. These findings highlight the complexity of ferritin’s function in cardiometabolic health, particularly in populations with unique dietary patterns like those in Mongolia.

Mongolian diets are distinctive in their high reliance on red meat, which provides significant amounts of dietary iron. This reliance may contribute to elevated ferritin levels, as observed in our study. Other studies of high-red-meat-consuming populations have similarly reported elevated ferritin due to increased iron intake [6,18,19,20]. However, unlike Western populations where ferritin is commonly associated with inflammation and CVD, our findings suggest that Mongolian adults may process dietary iron differently, possibly due to genetic or metabolic adaptations. This phenomenon has been observed in Inuit populations, where genetic adaptations allow for the effective metabolism of high-fat, high-protein diets with minimal cardiovascular impact [21].

Our study also raises questions about the utility of ferritin as a universal biomarker for CVD risk and inflammation across diverse populations. Ferritin is frequently linked to oxidative stress and inflammation in individuals with obesity and type 2 diabetes [8,9,10,11,12]. However, in this study, we found no significant association between ferritin and inflammatory markers such as CRP and IL-6, suggesting that ferritin may not reflect inflammation in this population. The levels of IL-6 observed in this study were indicative of chronic inflammation typical of T2DM. Similarly, the CRP levels aligned with findings from other populations, supporting the relevance of these markers in understanding the inflammatory profile of T2DM individuals. A study by Kado et al. [22] reported that serum IL-6 levels in patients with type 2 diabetes mellitus (T2DM) were significantly higher than in healthy controls, indicating a state of chronic inflammation typical of T2DM. Similarly, Vinagre et al. [23] found that C-reactive protein (CRP) levels in T2DM patients were elevated, aligning with our findings. The differences in body mass index (BMI) between these studies and ours, where the mean BMI did not exceed 30, may partially explain the variations in inflammatory marker levels. Although ferritin levels in our population did not directly correlate with cardiovascular disease risk or inflammatory markers, the overall inflammatory profile remained characteristic of T2DM. Our finding contrasts with results from studies of Western populations, where ferritin often correlates with systemic inflammation and is considered a CVD risk marker [9,10,11]. These differences highlight the importance of developing population-specific health markers that account for dietary, environmental, and genetic factors.

Our findings on the association between ferritin and LDL cholesterol suggest a potential role of ferritin in lipid metabolism, particularly among diabetic individuals. Ferritin has been associated with lipid abnormalities and poor glycemic control in various studies, linking it to pathways involved in lipid synthesis and glucose homeostasis [24,25]. However, the strength of this association was modest in our study, indicating that ferritin may not fully explain lipid abnormalities in Mongolian adults. These results underscore the complexity of metabolic interactions between diet and health markers in different populations, where high meat intake might impact lipid profiles without increasing systemic inflammation.

The findings from this study suggest the need for culturally adapted dietary guidelines that consider unique dietary patterns and genetic factors. In Western populations, high red meat intake is often discouraged due to its association with CVD risk, but, for Mongolians, a nuanced approach may be more appropriate to avoid nutritional deficits while minimizing potential health risks. Current global guidelines may not fully apply to populations with traditional, high-meat diets such as Mongolians. This study supports the need for population-specific dietary recommendations that account for cultural practices, environmental conditions, and genetic adaptations. Furthermore, the observation that 56% of participants did not adhere to a diabetes-specific diet highlights the challenges of dietary compliance in diabetes management and could partly explain the variability in ferritin and cardiovascular risk markers.

While red meat consumption is a distinguishing feature of the Mongolian diet, other dietary differences must also be considered. The intake of processed foods and high-density carbohydrates remains relatively low in Mongolia compared to Western countries, where such foods significantly contribute to the prevalence of CVD and T2D [26,27]. Additionally, fat consumption patterns, including the types of fat consumed, may vary and influence cardiovascular and metabolic health [28]. These differences highlight the need for further research into how the unique dietary patterns of Mongolian populations impact CVD and diabetes risk.

Menopausal status, which can influence ferritin levels and cardiovascular markers, ref. [10] was not collected as a variable in this study. This omission represents a limitation, as hormonal changes during menopause can significantly affect these markers. Future studies should include menopausal status to better understand its role in ferritin variability and its association with cardiovascular risk. Furthermore, subgroup analyses specifically focusing on younger participants were not conducted due to the limited number of individuals in this age group. While the overall correlations observed in this study appeared consistent across age groups, future studies may benefit from targeted analyses to explore age-specific trends in greater detail.

Future research should investigate the genetic and epigenetic factors in Mongolian populations that may affect how high meat intake influences health outcomes. Longitudinal studies are essential to determine if elevated ferritin levels in this population are linked to adverse cardiovascular or metabolic outcomes over time or if ferritin primarily reflects dietary habits. Advanced molecular analyses may identify biomarkers that more accurately reflect health risks associated with unique dietary patterns in Mongolia and similar populations. Although seasonal dietary variations could have influenced the results, 78.9% of participants reported no significant changes in their meat intake across seasons. This stability in dietary patterns may reflect urbanization trends in Mongolia, where access to meat is less seasonally dependent. Future studies should consider seasonal influences in greater detail.

## 5. Conclusions

In this diabetic Mongolian population, high meat intake was associated with elevated ferritin levels, which may have reflected dietary iron intake rather than systemic inflammation or increased CVD risk. These findings suggest that ferritin may function differently as a biomarker in populations with distinct dietary patterns and underscore the need for culturally specific dietary and health guidelines.

## Figures and Tables

**Table 1:** Baseline characteristics of participants across meat intake categories.

Variables	Meat Intake Category				*p*-Value			
	Total (n = 171)	T1, Low (n = 59)	T2, Middle (n = 59)	T3, High (n = 53)	Overall	T1 vs. T2	T2 vs. T3	T1 vs. T3
Age (year)	56.68 ± 10.51	58.35 ± 10.15	55.08 ± 10.9	56.6 ± 10.37	0.240	0.09	0.45	0.36
Gender, male, n (%)	63 (36.8%)	22 (37.3%)	22 (37.3%)	19 (35.8%)	0.984	1	0.87	0.87
Education (below bachelor) n (%)	103 (60.3%)	38 (64.5%)	31 (52.6%)	34 (64.2%)	0.245	0.19	0.21	0.97
Married or cohabiting, n (%)	131 (76.6%)	41 (69.5%)	49 (83.1%)	41 (77.4%)	0.218	0.08	0.44	0.34
Smoking, yes, n (%)	42 (24.6%)	16 (27.1%)	13 (22%)	13 (24.5%)	0.814	0.52	0.75	0.75
Alcohol, yes, n (%)	45 (30.4%)	12 (26.1%)	12 (23.5%)	21 (41.2%)	0.114	0.77	0.05	0.11
Diet, no, n (%)	94 (56%)	28 (49.1%)	37 (63.8%)	29 (54.7%)	0.278	0.11	0.33	0.55
Exercise, no, n (%)	60 (35.3%)	19 (32.8%)	20 (33.9%)	21 (39.6%)	0.641	0.59	0.53	0.49
Complication, n (%)	70 (41%)	28 (47.5%)	21 (35.6%)	21 (39.6%)	0.826	0.19	0.66	0.40
Duration of diabetes	9.63 ± 7.19	10.07 ± 8.11	8.68 ± 6.71	9.49 ± 6.57	0.317	0.93	0.45	0.57

Data are presented as mean ± SD or mean (minimum to maximum) and number (percentages, %). Note: SD, standard deviation.

**Table 2:** Biochemical and clinical parameters across meat intake categories.

Variables	Meat Intake Category				*p*-Value			
	Total (n = 171)	T1, Low (n = 59)	T2, Middle (n = 59)	T3, High (n = 53)	Overall	T1 vs. T2	T2 vs. T3	T1 vs. T3
Meat intake max	227.6 ± 94.62	163.89 ± 54.29	234.74 ± 89.68	290.56 ± 91.15	**0.001**	**0.001**	**0.001**	**0.001**
Meat intake mean 3 days	136.57 ± 71.54	64.97 ± 19.54	132.76 ± 26.21	220.5 ± 50.27	**0.001**	**0.001**	**0.001**	**0.001**
BMI	29.62 ± 4.98	29.63 ± 5.42	29.88 ± 4.5	29.36 ± 5.1	0.869	0.805	0.58	0.79
SPB (mm.Hg)	128.86 ± 14.22	128.74 ± 13.65	128.74 ± 11.21	129.13 ± 17.69	0.986	0.99	0.88	0.89
DPB (mm.Hg)	84.55 ± 11.17	85.76 ± 11.4	84.56 ± 10.11	83.31 ± 12.1	0.582	0.59	0.58	0.32
HbA1c (%)	8.73 ± 2.45	8.79 ± 2.53	8.71 ± 2.42	8.69 ± 2.43	0.976	0.86	0.97	0.84
Total cholesterol (mmol/L)	5.37 ± 1.12	5.38 ± 1.12	5.32 ± 0.99	5.43 ± 1.27	0.878	0.77	0.61	0.81
TG (mmol/L)	2.08 ± 1.51	2.13 ± 1.4	2.1 ± 1.97	2.01 ± 1.13	0.925	0.92	0.79	0.64
HDL (mmol/L)	1.25 ± 0.22	1.26 ± 0.21	1.27 ± 0.2	1.24 ± 0.24	0.75	0.77	0.46	0.64
LDL (mmol/L)	3.43 ± 0.98	3.41 ± 0.94	3.35 ± 0.95	3.51 ± 1.05	0.749	0.8	0.47	0.61
RBC	4.91 ± 0.52	4.81 ± 0.47	4.99 ± 0.6	4.95 ± 0.48	0.278	0.14	0.74	0.19
HGB	14.22 ± 1.66	13.99 ± 1.61	14.39 ± 1.93	14.31 ± 1.46	0.503	0.302	0.82	0.33
HCT	41.75 ± 4.52	41.2 ± 4.23	42.08 ± 5.26	42.02 ± 4.13	0.61	0.404	0.95	0.36
Ferritin	241.9 ± 201.9	119.6 ± 172.1	254.0 ± 224.2	275.6 ± 202.1	0.118	0.14	0.59	0.34
Homocysteine	11.76 ± 5.17	11.86 ± 5.72	12.02 ± 5.18	11.37 ± 4.64	0.851	0.89	0.56	0.68
IL-6	4.36 ± 5.32	3.82 ± 3.71	4.99 ± 6.66	4.25 ± 5.21	0.484	0.23	0.51	0.61
CRP	1.2 ± 4.38	2 ± 7.08	0.87 ± 1.82	0.68 ± 1.32	0.218	1.33	0.29	0.58
sTFR	14.83 ± 6.87	14.49 ± 4.93	14.88 ± 5.71	15.13 ± 9.47	0.895	0.705	0.86	0.66
RET He	28.4 ± 2.08	28.42 ± 2.17	28.11 ± 2.52	28.68 ± 1.37	0.397	0.49	0.16	0.48
Total FRS	12.43 ± 4.41	12.74 ± 4.41	11.89 ± 4.57	12.69 ± 4.25	0.511	0.304	0.34	0.95

Data are presented as mean ± SD or mean (minimum to maximum) and number (percentages, %). Bold values denote statistical significance at the *p* < 0.05 level. Note: SD, standard deviation.

**Table 3:** Comparison of clinical and biochemical characteristics by ferritin level categories in diabetic individuals.

Variables	Ferritin Category			*p* Value
	Total (n = 171)	Normal (n = 102)	Elevated (n = 69)	
Age (year)	56.68 ± 10.51	55.97 ± 11.4	57.7 ± 9	0.282
Gender, male, n (%)	63 (36.8%)	49 (48%)	14 (20.3%)	**0.0001**
Education (below bachelor) n (%)	103 (60.3%)	57 (55.9%)	46 (66.7%)	0.456
Married or cohabiting, n (%)	131 (76.6%)	82 (80.4%)	49 (71%)	0.197
Smoking, yes, n (%)	42 (24.6%)	28 (27.5%)	14 (20.3%)	0.366
Alcohol, yes, n (%)	45 (30.4%)	29 (33%)	16 (26.7%)	0.469
Diet, no, n (%)	94 (56%)	57 (57%)	37 (54.4%)	0.754
Exercise, no, n (%)	60 (35.3%)	35 (34.3%)	25 (36.8%)	0.687
Complication, number, n (%)	70 (40.9%)	43 (42.2%)	27 (39.1%)	0.745
Duration of diabetes	9.63 ± 7.19	10.2 ± 7.68	8.7 ± 6.33	0.199
Meat intake max	227.6 ± 94.62	223.72 ± 91.95	233.33 ± 98.84	0.516
Meat intake mean 3 days	136.57 ± 71.54	131.24 ± 69.53	144.44 ± 74.22	0.238
BMI	29.62 ± 4.98	29.7 ± 4.86	29.51 ± 5.19	0.817
SPB (mm.Hg)	128.86 ± 14.22	128.89 ± 15.91	128.82 ± 11.38	0.974
DPB (mm.Hg)	84.55 ± 11.17	84.67 ± 11.39	84.37 ± 10.95	0.877
HbA1c	8.73 ± 2.45	8.6 ± 2.42	8.9 ± 2.5	0.411
Total cholesterol (mmol/L)	5.37 ± 1.12	5.2 ± 0.94	5.63 ± 1.31	**0.012**
TG (mmol/L)	2.08 ± 1.51	2.06 ± 1.24	2.11 ± 1.87	0.849
HDL (mmol/L)	1.25 ± 0.22	1.23 ± 0.18	1.29 ± 0.26	0.133
LDL (mmol/L)	3.43 ± 0.98	3.22 ± 0.84	3.75 ± 1.09	**0.002**
RBC	4.91 ± 0.52	4.94 ± 0.55	4.87 ± 0.47	0.455
HGB	14.22 ± 1.66	14.1 ± 1.77	14.3 ± 1.49	0.478
HCT	41.75 ± 4.52	41.5 ± 4.69	42 ± 4.22	0.618
Homocysteine	11.76 ± 5.17	12.42 ± 5.43	10.68 ± 4.55	0.077
IL-6	4.36 ± 5.32	4.35 ± 5.39	4.36 ± 5.27	0.995
CRP	1.2 ± 4.38	0.8 ± 1.82	1.79 ± 6.5	0.147
sTFR	14.83 ± 6.87	14.71 ± 6	15.02 ± 8.05	0.778
RET He	28.4 ± 2.08	28.05 ± 2.49	28.87 ± 1.23	**0.016**
Total FRS	12.43 ± 4.41	11.4 ± 4.32	13.97 ± 4.1	**0.0001**

Data are presented as mean ± SD or mean (minimum to maximum) and number (percentages, %). Bold values denote statistical significance at the *p* < 0.05 level. Note: SD, standard deviation.

## Data Availability

The data used to support the findings of this study are available from the corresponding author upon request. The data are not publicly available due to privacy and ethical reasons.
